# Supplementary material for: Long-range inhibition synchronizes and updates prefrontal task activity
Source: Nature. 2023 Apr 26;617(7961):548–54. doi: 10.1038/s41586-023-06012-9 (PMC10191848; doi:10.1038/s41586-023-06012-9)
Supplement: Supplementary file 2 — Reporting Summary [file 41586_2023_6012_MOESM2_ESM.pdf]

nature research

Corresponding author(s): Vikaas S. Sohal

Last updated by author(s): Mar 8, 2023

Reporting Summary

Nature Research wishes to improve the reproducibility of the work that we publish. This form provides structure for consistency and transparency in reporting. For further information on Nature Research policies, see Authors & Referees and the Editorial Policy Checklist.

Statistics

For all statistical analyses, confirm that the following items are present in the figure legend, table legend, main text, or Methods section.

n/a	Confirmed	
☐	☒	The exact sample size (*n*) for each experimental group/condition, given as a discrete number and unit of measurement
☐	☒	A statement on whether measurements were taken from distinct samples or whether the same sample was measured repeatedly
☐	☒	The statistical test(s) used AND whether they are one- or two-sided<br>*Only common tests should be described solely by name; describe more complex techniques in the Methods section.*
☐	☒	A description of all covariates tested
☐	☒	A description of any assumptions or corrections, such as tests of normality and adjustment for multiple comparisons
☐	☒	A full description of the statistical parameters including central tendency (e.g. means) or other basic estimates (e.g. regression coefficient) AND variation (e.g. standard deviation) or associated estimates of uncertainty (e.g. confidence intervals)
☐	☒	For null hypothesis testing, the test statistic (e.g. *F*, *t*, *r*) with confidence intervals, effect sizes, degrees of freedom and *P* value noted<br>*Give P values as exact values whenever suitable.*
☒	☐	For Bayesian analysis, information on the choice of priors and Markov chain Monte Carlo settings
☒	☐	For hierarchical and complex designs, identification of the appropriate level for tests and full reporting of outcomes
☒	☐	Estimates of effect sizes (e.g. Cohen's *d*, Pearson's *r*), indicating how they were calculated

Our web collection on statistics for biologists contains articles on many of the points above.

Software and code

Policy information about availability of computer code

Data collection	Synapse 84 (Tucker-Davis Technologies), nVoke2 (Inscopix, Inc.), ANY-maze (Stoelting Europe), pClamp (Molecular Devices).
Data analysis	Statistical analyses were performed in Matlab (Mathworks) and Prism 8 (GraphPad). OpenScope (Tucker-Davis Technologies) was used for behavioral scoring. Image processing was performed using ImageJ. Adobe Illustrator was used for assembling figures.

For manuscripts utilizing custom algorithms or software that are central to the research but not yet described in published literature, software must be made available to editors/reviewers. We strongly encourage code deposition in a community repository (e.g. GitHub). See the Nature Research guidelines for submitting code & software for further information.

Data

Policy information about availability of data

All manuscripts must include a data availability statement. This statement should provide the following information, where applicable:

- Accession codes, unique identifiers, or web links for publicly available datasets
- A list of figures that have associated raw data
- A description of any restrictions on data availability

The data that support the findings of this study are available from the corresponding author upon reasonable request. The underlying physiological data (trial-by-trial measurements of synchrony and population activity vectors) and associated MATLAB code are available on Zenodo at https://doi.org/10.5281/zenodo.7709805.

Field-specific reporting

Please select the one below that is the best fit for your research. If you are not sure, read the appropriate sections before making your selection.

☒ Life sciences ☐ Behavioural & social sciences ☐ Ecological, evolutionary & environmental sciences

For a reference copy of the document with all sections, see nature.com/documents/nr-reporting-summary-flat.pdf

Life sciences study design

All studies must disclose on these points even when the disclosure is negative.

Sample size	No statistical methods were used to predetermine sample size, but sample size choice was based on previous studies (Cho et al., 2015) and are consistent with those generally employed in the field.
Data exclusions	In all behavioral experiments (slice electrophysiology, optogenetics, dual-site voltage-indicator photometry, in vivo calcium imaging), we verified that viral expression and/or fiber tip placement was in the target structure. No data was excluded.
Replication	All key findings were replicated in new cohorts, and all attempts at replication were successful.
Randomization	Animals were randomly assigned numbers and tested blind for the experimental condition.
Blinding	All behavioral experiments were performed, scored, and analyzed blind to the virus injected.

Reporting for specific materials, systems and methods

We require information from authors about some types of materials, experimental systems and methods used in many studies. Here, indicate whether each material, system or method listed is relevant to your study. If you are not sure if a list item applies to your research, read the appropriate section before selecting a response.

Materials & experimental systems

n/a	Involved in the study	
☒	☐	Antibodies
☒	☐	Eukaryotic cell lines
☒	☐	Palaeontology
☐	☒	Animals and other organisms
☒	☐	Human research participants
☒	☐	Clinical data

Methods

n/a	Involved in the study	
☒	☐	ChIP-seq
☒	☐	Flow cytometry
☒	☐	MRI-based neuroimaging

Animals and other organisms

Policy information about studies involving animals; ARRIVE guidelines recommended for reporting animal research

Laboratory animals	Male and female mice of the following C57/Bl6 strains were used, at 10-25 weeks of age: PV-Cre, Ai14 (all from The Jackson Laboratory).
Wild animals	The study did not involve wild animals.
Field-collected samples	The study did not involve samples collected from the field.
Ethics oversight	All animal care, procedures, and experiments were conducted in accordance with the NIH guidelines and approved by the Administrative Panels on Laboratory Animal Care at the University of California, San Francisco as well as followed French and European guidelines for animal experimentation and in compliance with the institutional animal welfare guidelines of the Paris Brain Institute.

Note that full information on the approval of the study protocol must also be provided in the manuscript.
